# Mouth opening variables amongst the North Indian population

**DOI:** 10.1016/j.jobcr.2024.11.003

**Published:** 2024-11-22

**Authors:** Disha Dixit, Pavitra Rastogi, Nand Lal, Rameshwari Singhal

**Affiliations:** Department of Periodontology, Faculty of Dental Sciences, King George's Medical University, U.P., Lucknow, India

**Keywords:** Mouth opening, Normal mouth opening, Maximum mouth opening, Mouth opening in Indian population

## Abstract

**Background:**

For the clinician to conveniently perform an extensive oral examination, it is required to have a known normal range of maximum mouth opening (MMO). This study aims to establish a standard for calculating the normal range of mouth opening in the adult population of North India. The results of this research will have clinical implications for the treatment and diagnosis of diseases that affect the mouth opening in adults, either directly or indirectly.

**Aim:**

To evaluate mean mouth opening in North Indian population and establish their correlation with age and gender.

**Method:**

662 adults from North Indian population, 317 males and 345 females in the age range of 18–80 years were evaluated on their consent. Maximum mouth opening was measured using calibrated vernier calliper scale. This study was conducted in the Department of Periodontology, Faculty of Dental Sciences, King George Medical University, Lucknow.

**Results:**

Males exhibited a higher mean MMO of 48.0 mm compared to females, who had a lower mean MMO of 45.1 mm among North Indian population of the country. In the statistical comparison of MMO between males and females within each age group, the results showed no significant differences.

**Conclusion:**

Mouth opening of females is less than males in all age groups and it seems to decrease with age in all age groups in both males and females. The variability of MMO with age appears statistically similar for both genders.

## Graphical abstract

**Figure:**
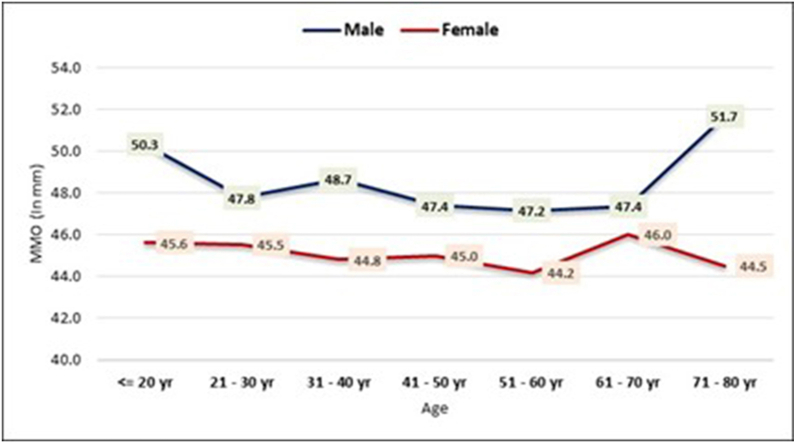

## 1 Introduction

Normal mouth opening is a crucial yet multifaceted concept, especially in a diverse population where variations abound in every aspect. It is essential to provide a detailed explanation of what constitutes “normal” within this context. Mouth opening serves not only as a significant diagnostic criterion for various oral diseases but also as a vital tool for clinicians, enabling them to effectively address oral structures. Moreover, it plays a pivotal role in determining treatment plans and significantly influences the prognosis of both oral hard and soft tissue diseases. Recognizing the intricacies of normal mouth opening is essential for accurate diagnosis and successful clinical outcomes in the realm of oral healthcare.

A decreased mouth opening is a critical sign of a progressive disease and can be considered as a marker to check the efficacy of treatment.^1^ Identifying its normal value or range is now essential in order to detect decreased mouth opening. The measurement of mouth opening exhibits substantial variation across demographic factors such as race, age, gender, and others.^2^ ^(3)^ Hence it becomes important to correlate these factors to what could be called as a normal mouth opening in a population.

The objective of this study is to investigate potential associations between gender and mouth opening in the research participants, as well as between mouth opening and age. By addressing these objectives collectively, the research endeavours to provide valuable insights into the relationship between mouth opening, and demographic factors, contributing to a broader understanding of oral health and well-being.

## 2 Materials and methods

The study was conducted in the Department of Periodontology, Faculty of Dental Sciences, King George's Medical University, Lucknow for 6 months from January 2024 to June 2024. Institutional ethical committee permission and patient consent were taken prior to commencement of the study. Sample size for the study was calculated to be 661 at 95 % confidence interval, considering 10 % attrition. Considering the same, the study included 662 adults from North Indian population, 317 males and 345 females in the age range of 18–80 years. All subjects provided informed consent for participation.

To analyze the decline in mouth opening with age, participants were divided into age groups with 10-year intervals (Table 2, Table 3). This approach generated a mean age for each group, enabling a more precise comparison of mouth opening across different age ranges.

Maximum mouth opening was measured when the subject was asked to comfortably sit in an upright position on a chair with a back rest^4^ and was asked to open their mouth wide open as much as possible. The examiner measured the maximum interincisal distance between the incisal edges of maxillary and mandibular central incisors at the midline with a standardised calibrated Vernier Calliper scale (Fig. I).^5^ Following data was collected from each subject:

1) UHID
2) Name
3) Age
4) Gender
5) Place
6) Mobile number
7) Maximum mouth opening

**Fig. 1:**
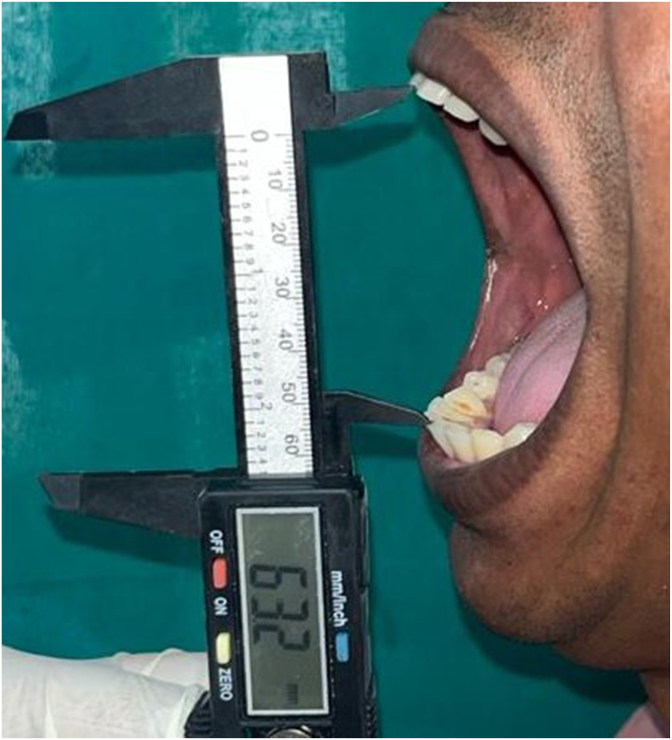
The examiner measured the maximum interincisal distance between the incisal edges of maxillary and mandibular central incisors at the midline with a standardised calibrated Vernier Calliper scale.

### 2.1 Inclusion criteria

1) Presence of functional maxillary and mandibular central incisors is required.
2) Normal positioning of anterior teeth is a key criterion.
3) Attrition on the incisal edges should not exceed 1 mm.
4) Participants must be 18 years of age or older.
5) Individuals should identify as of North Indian origin.

### 2.2 Exclusion criteria

1) Individuals with a history of jaw or face pain either at rest or during function will not be considered.
2) Those with a history of bruxism, temporomandibular disorders (TMDs), malignancy, or exhibiting facial or dental developmental abnormalities will be excluded.
3) Individuals with dental prostheses on their anterior teeth are not eligible for participation.

### 2.3 Observations

The study examined the relationship between Maximum Mouth Opening (MMO) and gender, providing insightful data on the mean MMO values for male and female participants. The results revealed a notable difference in mean MMO values between the two genders (Table I, Fig. II). Specifically, males exhibited a higher mean MMO of 48.0 mm compared to females, who had a lower mean MMO of 45.1 mm. Statistical analysis using an independent samples *t*-test yielded a t-value of 5.13 with a highly significant p-value of less than 0.001. This indicates a significant association between sex and Maximum Mouth Opening, suggesting that males, on average, have a larger MMO compared to females in the study population.

**Table 1:** The results revealed a notable difference in mean Maximum Mouth Opening values between the two genders.

Gender	MMO (In mm)	
	Mean	SD
Male	48.0	7.7
Female	45.1	6.7
Significance	p < 0.001∗

**Fig. 2:**
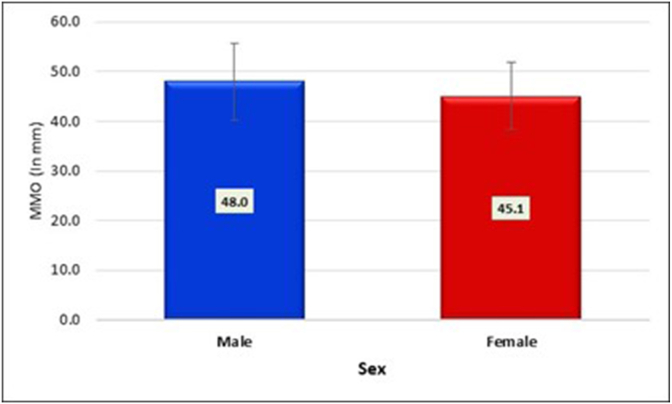
Notable difference in mean Maximum Mouth Opening values between the two genders.

The study also investigated the association between Maximum Mouth Opening (MMO) and age, providing elaborate data on the mean MMO values across different age groups (Table II, Fig. III). The results indicate a slight variation in mean MMO values across age categories. Specifically, participants aged 71–80 years demonstrated the highest mean MMO of 51.1 mm, while those aged 51–60 years showed the lowest mean MMO at 45.8 mm. However, the overall analysis using a one-way ANOVA test yielded an F-value of 1.09 with a corresponding p-value of 0.366. The non-significant p-value suggests that there is no statistically significant association between age and Maximum Mouth Opening among the study participants.

**Table 2:** The mean Maximum Mouth Opening (MMO) values across different age groups.

Age	MMO (In mm)	
	Mean	SD
≤ 20 yr	47.0	9.4
21–30 yr	46.5	7.4
31–40 yr	46.5	7.6
41–50 yr	46.3	5.9
51–60 yr	45.8	6.9
61–70 yr	46.8	8.3
71–80 yr	51.1	5.9
significance	F = 1.09, p = 0.366

**Fig. 3:**
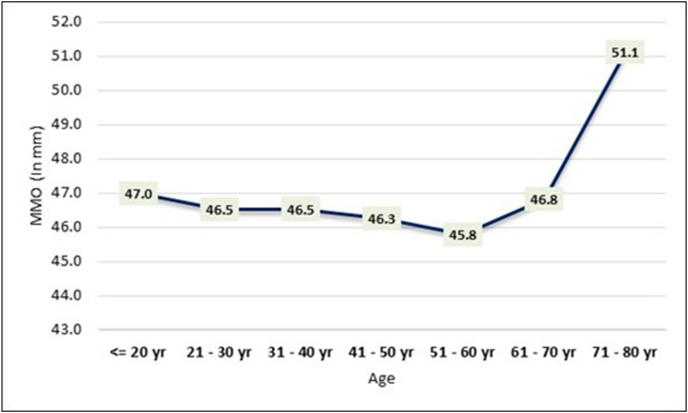
Mean Maximum Mouth Opening values across different age groups.

The analysis of Maximum Mouth Opening (MMO) in millimeters (mm) among different age groups and genders revealed interesting findings. For males, the mean MMO in the age groups varied, with the highest mean observed in the 71–80 years age group (51.7 mm), while for females, the highest mean was in the≤20 years age group (45.6 mm). Standard deviations (SD) indicated the degree of variability within each subgroup.

In the statistical comparison of MMO between males and females within each age group, the results showed no significant differences (Table III). The F-statistic and p-values for each age group demonstrated that the MMO means were not significantly different between genders. Specifically, for males, the F-value was 0.97 with a p-value of 0.443, and for females, the F-value was 0.33 with a p-value of 0.923.

**Table 3:** In the statistical comparison of Maximum mouth opening (MMO) between males and females within each age group, the results showed no significant differences.

MMO (In mm)	Male		Female	
	Mean	SD	Mean	SD
≤ 20 yr	50.3	9.7	45.6	9.0
21–30 yr	47.8	8.5	45.5	6.3
31–40 yr	48.7	8.1	44.8	6.7
41–50 yr	47.4	6.2	45.0	5.3
51–60 yr	47.2	7.0	44.2	6.4
61–70 yr	47.4	8.7	46.0	8.0
71–80 yr	51.7	5.8	44.5	
significance	F = 0.97, p = 0.443		F = 0.33, p = 0.923	

These findings suggest that, based on the MMO measurements, there is no significant gender-based difference with increasing age. It's important to note the variability in MMO across age groups and genders, providing valuable insights into the distribution of maximum mouth opening measurements within the studied population.

## 3 Discussion:

Maximum Mouth opening has been described by Agerberg, 1974 as the maximum interincisal distance, or corrected interincisal distance where vertical overlap between the incisors is added to it.^6^ However, an active mouth opening achieved by the patient without assistance can be considered as clinically relevant. Wood & Branco in 1979 have reported various methods of measuring interincisal distance and concluded that direct measurement was the most accurate.^5^

The results of this study reveal that the mean maximum mouth opening for males is 48.0 mm, while for females, it is 45.1 mm. These measurements align with similar studies conducted in different populations. For instance, a study focusing on the adult Irish population found that males had a mean MMO of 43.3 mm, and females had 41.4 mm.^2^ Additionally, Cox and Walker's investigation on 700 Nepalese adults reported a mean interincisal distance of 47.1 mm.^7^ These results agree with another classic study, where the average maximal mouth opening for males was 51.3 mm (SD 8.3), with a range of 39–70 mm and for females, the average maximal mouth opening was 44.3 mm (SD 6.7), with a range of 36–56 mm.^8^

Existing research consistently indicates a decline in mouth opening among adults as they age.^2^ In the current study, the data suggests that the mean mouth opening for individuals aged 21–30 years (constituting 28.7 % of subjects) is 46.5 mm, for those aged 41–50 years (18.6 % of subjects) it is 46.3 mm, and for the 51–60 years age group (14.4 % of subjects), the mean value is 45.8 mm. Although the mean mouth opening for the 61–70 years age group is 46.8 mm and for the 71–80 years age group it is 51.1 mm, these values are deemed statistically non-significant as they account for only 14.4 % and 6.8 % of subjects, respectively. Consequently, the collective data implies a decrease in mean mouth opening with age for both genders. A similar study showed a gradual decrease in mouth opening with age. In the 21–30 year group, the mean mouth opening was 56.7 mm for males and 51.7 mm for females. For the 31–40 year group, it was 57.1 mm for males and 50.7 mm for females. In the 41–50 year group, it decreased to 52.5 mm for males and 47.7 mm for females. Among 51–60 year olds, it was 48.3 mm for males and 42.0 mm for females, and in the 61–70 year group, it further reduced to 45.8 mm for males and 40.2 mm for females.^8^ Nutritional decline is a major factor for oral frailty with increasing age and that could also be a cause for such inference.^9^ No other association with nutrition was found in evidence.

The data presented in Table III illustrates that, across all age groups, mean mouth opening is higher in males compared to females. Specifically, for the age group of 21–30 years, males exhibit an MMO of 47.8 ± 8.5, while females show 45.5 ± 6.3. In the 41–50 years age group, males have an MMO of 47.4 ± 6.2, and females have 45.0 ± 5.3. Similarly, in the 71–80 years age group, males demonstrate an MMO of 51.7 mm, while females have 44.5 mm. Despite these variations, the findings indicate that the decrease in MMO measurements with age does not result in a significant gender-based difference, as depicted in the accompanying chart and graph. The variability of MMO with age appears statistically similar for both genders.

Upon reviewing studies on mouth opening, it is concluded that, in general and within specific age groups, females tend to have a smaller mean mouth opening compared to males. This could be attributed to a longer mandibular length or greater muscle strength, allowing males to open their mouths wider. Also, other reasons could be the fact that the anatomical structure of the head and facial bones in males is generally larger than that in females.^3^^,^^8^ However, Pullinger et al. reported that maximum mouth opening (MMO) was greater in females than in males. This aligns with the broader understanding that females typically exhibit greater joint mobility than males. Research on temporomandibular joint (TMJ) mobility, angle of mouth opening, and joint laxity collectively suggests that women generally have a wider range of mouth opening motion compared to men..^10^^,^^11^

There are few limitations of the study like it does not include patients below 21 years of age and it only involves patients of North Indian region.

## 4 Conclusion

Mean of Maximum Mouth opening for males is 48.0 mm with a range of 40.3 mm–55.7 mm and for females is 45.1 mm with a range of 38.4 mm–51.8 mm. This concludes that mouth opening of females is significantly less than males in all age groups. Also, mouth opening declines with age for both males and females.

## Informed consent

Written informed consent form were signed and taken from all the participants.

## Ethics approval and consent to participate

Approved from Institutional Ethics Committee and Written Informed consent taken.

## Sources of funding

This research did not receive any specific grant from funding agencies in the public, commercial, or not-for-profit sectors.

The research was self funded and no other sources of funding are involved in any part of the project or manuscript writing.

## Declaration of competing interest

The authors declare that they have no known competing financial interests or personal relationships that could have appeared to influence the work reported in this paper.
